# Editorial: The future of bio-inspired robotics: an early career scientists’ perspective

**DOI:** 10.3389/frobt.2024.1370948

**Published:** 2024-02-13

**Authors:** Marcello Calisti, Li Wen

**Affiliations:** ^1^ Lincoln Institute for Agri-Food Technology, University of Lincoln, Lincoln, United Kingdom; ^2^ School of Mechanical Engineering and Automation, Beihang University, Beijing, China

**Keywords:** bioinspired robotics, soft robotics, robot simulation, early career scientists, soft 7 robotics, robot design

Humans have attempted to mimic nature’s solutions for over 4,000 years, but modern bioinspired methodologies have their roots and formalizations in the ’50s. Since then, bioinspiration fostered innovation in diverse aspects of our technologies, from software to hardware solutions. Bioinspired robots are ubiquitous today, and research efforts are directed toward disparate fields, including but not limited to applications like environmental conservation, medicine, exploration, defense, and fundamental research. Bioinspired methodologies can serve as more effective technological tools for various tasks and provide an investigation alternative to unveil nature’s secrets via technical counterparts. With this impressive range of scope and opportunities, this Research Topic focuses on the contributions of early career scientists and their point of view on the field they will contribute to advocating and shaping. Even if brief, the Research Topic covered vital aspects and the complexity of the field, providing a reflection point on the future of bioinspiration.

For example, (Angelidis) illustrates the challenges and the need for robust and comprehensive simulation environments. The multi-disciplinary nature of bioinspiration requires an unprecedented effort toward integrating simulation with multiple physics, easily extendable and modular to include the needed details and novel discoveries. The granularity of simulation, and therefore the detail of bioinspiration, is also covered and reflected by (Ren and Shao), who explained their point of view on “delicate structure” and how a bottom-up approach might benefit the future of bioinspiration. Again, it is highlighted how those low-level components interact with each other and how they have the potential to explore deformations, plasticity, and control to create the emergence of behaviors. This concept also underpins the research on Soft Robotics and how the body can be exploited for computation, safe interaction, and sustainability. Therefore, it is not surprising that this Research Topic is explored in detail in the reflection of (Tauber and Slesarenko), where the pros and cons of soft robotics concerning control, energy autonomy, sustainability and compliance are analyzed to reflect on how the field could move forward. Eventually, an emerging and intriguing application of bioinspired robots underlies in their potential as conservation tools. Presented by (Chellapurath et al.), it is exciting to see how bioinspired robots are already venturing into the field and providing effective alternatives for exploration, and as well it is essential to reflect on the next steps that would make the solutions more robust, efficient, and effective.

This Research Topic succinctly illustrated this field’s complexity and potential, and it shed light on the possible future of bioinspired research, that will be pioneered by the Early Career Scientists of our field.

